# S66. THEORY OF MIND IN A FIRST-EPISODE PSYCHOSIS POPULATION USING THE HINTING TASK

**DOI:** 10.1093/schbul/sby018.853

**Published:** 2018-04-01

**Authors:** Maija Lindgren, Minna Torniainen-Holm, Inkeri Heiskanen, Greta Voutilainen, Ulla Pulkkinen, Tuukka Mehtälä, Markus Jokela, Tuula Kieseppä, Jaana Suvisaari, Sebastian Therman

**Affiliations:** 1 National Institute for Health and Welfare; 2 Helsinki Deaconess Institute; 3 Social Services and Health Care, City of Helsinki, Aurora Hospital; 4 National Institute for Health and Welfare, Royal College of Surgeons in Ireland; 5 Hospital District of Helsinki and Uusimaa, Hyvinkää Hospital Area; 6 Helsinki University; 7 National Institute for Health and Welfare, University of Helsinki and Helsinki University Hospital

## Abstract

**Background:**

Impaired social functioning is one of the most apparent features of psychotic disorders. Deficits in social cognition may explain impaired functioning even more than the other cognitive deficits related to psychosis. Of the areas of social cognition, especially relevant to psychosis appear to be deficiencies in theory of mind (ToM), the ability to perceive and interpret the mental states of others, or “mentalizing”. Currently, it is unclear to what extent general cognitive deficits explain impairment in ToM.

We wanted to explore 1) the possible difference in ToM between first-episode psychosis (FEP) patients and controls, 2) whether diagnosis group (schizophrenia vs. other psychotic disorders) and level of functioning are associated with ToM, and 3) to what extent these differences are explained by general cognitive performance.

**Methods:**

This study examined ToM in young adults with FEP (n=66). Of those, 25 had schizophrenia and the rest were diagnosed with other psychotic disorders. Age- and gender-matched controls were identified from the Population Information System (n=62). The participants were administered a broad neuropsychological assessment, part of which was the Hinting Task assessing ToM. With 10 short discussions in everyday situations, the Hinting Task assesses the ability to conclude, from indirect speech, what another person really means.

A factor score of the Hinting Task was formed, taking into account the varying difficulty level and relevance of the 10 items. To investigate the association between ToM and general cognitive functions, we summarized non-social cognitive performance constructing a “g factor”, which was used as an overall index of general cognitive performance.

**Results:**

The internal consistency of the Hinting Task calculated from the dichotomized data was modest, with McDonald’s categorical omega estimated at .57 (95 % CI .36, .71). In the single dimensional factor solution, items 8 and 9 had the weakest loadings and item 10 the strongest. Items 9 and 10 of the Hinting Task were the easiest, and items 1 and 8 were the most difficult.

Participants with FEP (mean score 16.0) performed worse than controls (mean score 17.4) on the Hinting Task (Cohen’s d=0.50 calculated from factor scores). However, the difference between FEP and control groups was no longer significant when general cognition was controlled for. 75 % of the variance between the groups was explained by general cognitive deficits, especially impaired processing speed (WAIS-III Digit Symbol) and episodic memory (WMS-III Logical Memory).

When the FEP group was divided according to diagnosis, those with schizophrenia scored lower on the Hinting Task than the other psychosis patients. The ToM difference between individuals with schizophrenia and controls (Cohen’s d=1.1) remained significant even when general cognitive performance was controlled for. In contrast, those with other psychotic disorders than schizophrenia did not differ from controls.

ToM performance of the best functioning patient group (20 patients who had a GAF score ≥50, Hinting Task mean score 17.8) did not differ from that of the control group.

**Discussion:**

Based on this study, and supporting previous findings (Bora & Pantelis, Schizophr. Res. 2013), deficits in ToM were already present in early psychosis. They were largely overlapping with deficits in general cognitive processes. However, and in line with previous meta-analytical findings (Sprong et al., Br. J. Psychiatry 2007), in a subgroup of the FEP patients with schizophrenia, impairments in ToM remained after controlling for overall cognitive functioning. In conclusion, specific deficits in ToM could be found in schizophrenia, independent from general cognitive deficits.

